# c-RET Molecule in Malignant Melanoma from Oncogenic RET-Carrying Transgenic Mice and Human Cell Lines

**DOI:** 10.1371/journal.pone.0010279

**Published:** 2010-04-21

**Authors:** Yuichiro Ohshima, Ichiro Yajima, Kozue Takeda, Machiko Iida, Mayuko Kumasaka, Yoshinari Matsumoto, Masashi Kato

**Affiliations:** 1 Units of Environmental Health Sciences, Department of Biomedical Sciences, College of Life and Health Sciences, Chubu University, Kasugai-shi, Aichi, Japan; 2 Department of Dermatology, Aichi Medical University School of Medicine, Nagakute-cho, Aichi-gun, Aichi, Japan; Dana-Farber Cancer Institute, United States of America

## Abstract

Malignant melanoma is one of the most aggressive cancers and its incidence worldwide has been increasing at a greater rate than that of any other cancer. We previously reported that constitutively activated RFP-RET-carrying transgenic mice (RET-mice) spontaneously develop malignant melanoma. In this study, we showed that expression levels of intrinsic c-Ret, glial cell line-derived neurotrophic factor (Gdnf) and Gdnf receptor alpha 1 (Gfra1) transcripts in malignant melanomas from RET-transgenic mice were significantly upregulated compared with those in benign melanocytic tumors. These results suggest that not only introduced oncogenic RET but also intrinsic c-Ret/Gdnf are involved in murine melanomagenesis in RET-mice. We then showed that c-RET and GDNF transcript expression levels in human malignant melanoma cell lines (HM3KO and MNT-1) were higher than those in primary cultured normal human epithelial melanocytes (NHEM), while GFRa1 transcript expression levels were comparable among NHEM, HM3KO and MNT-1. We next showed c-RET and GFRa1 protein expression in HM3KO cells and GDNF-mediated increased levels of their phosphorylated c-RET tyrosine kinase and signal transduction molecules (ERK and AKT) sited potentially downstream of c-RET. Taken together with the finding of augmented proliferation of HM3KO cells after GDNF stimulation, our results suggest that GDNF-mediated c-RET kinase activation is associated with the pathogenesis of malignant melanoma.

## Introduction

It has recently been reported that the incidence of cutaneous malignant melanoma is increasing at a greater rate than that of any other cancer [1]. Since malignant melanoma is known as one of the most aggressive human cancers, malignant melanoma is one of threats for public health.

**Figure 1:**
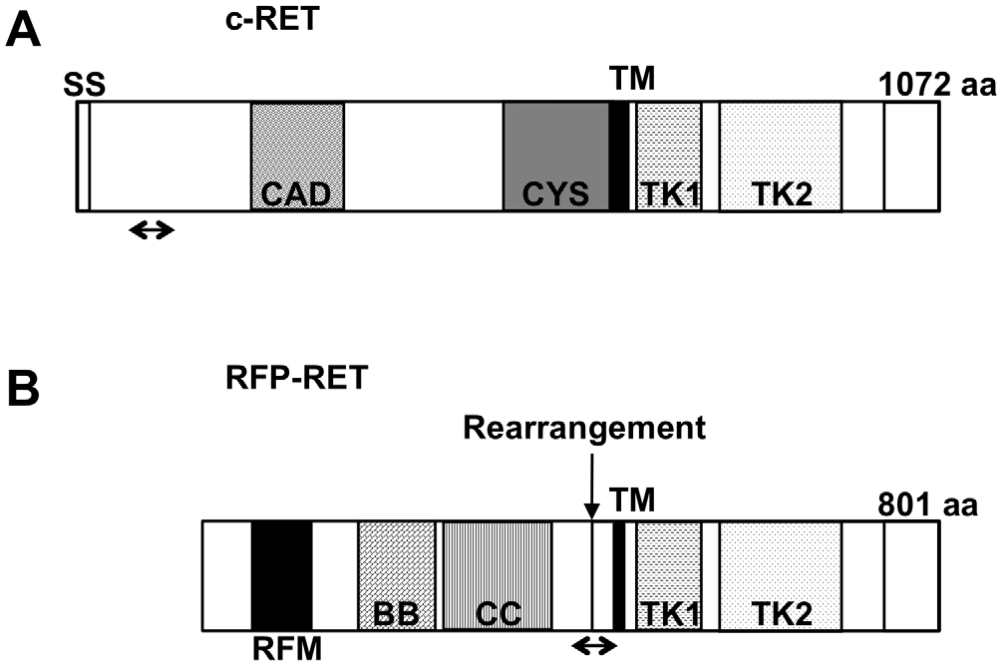
Scheme of c-RET and RFP-RET cDNA constructs. (A) c-RET cDNA. (B) RFP-RET cDNA. The sites of c-RET (A) and RFP-RET (B) primers for real-time PCR analysis are shown by arrows. SS, signal sequence; CAD, cadherinlike domain; CYS, cysteine-rich region; TM, transmembrane domain; TK1, tyrosine kinase domain 1; TK2, tyrosine kinase domain 2; aa, amino acids; RFM, RING finger motif; BB, B box; CC, coiled coil.

The c-RET proto-oncogene (Figure 1A) encodes a receptor-tyrosine kinase [2]. Previously, glial cell line-derived neurotrophic factor (GDNF) has been reported to be one of ligands for c-RET [2]. GDNF, which is structurally related to members of the transforming growth factor- ß (TGF-ß) superfamily, exerts its effect on target cells by binding to a glycosyl phosphatidylinositol (GPI)-anchored cell surface protein (GFRa1), which, in turn, recruits the receptor tyrosine kinase c-RET to form a multi-subunit signaling complex. Formation of this complex results in c-RET autophosphorylation and a cascade of intracellular signaling including the extracellular signal-regulated kinase (ERK) and Akt kinase to regulate cell survival [2]–[6]. Results of our previous study suggest that autophosphorylation of tyrosine 905 in c-RET is crucially important for its kinase and transforming activity [7]. On the other hand, it has been shown that mutationally enhanced activity of c-RET kinase caused the development of human carcinomas, including multiple endocrine neoplasia (MEN) and papillary thyroid carcinoma (PTC) [4], [8].

RFP-RET (Figure 1B) is a hybrid oncogene between c-RET and RFP that was isolated by NIH3T3 transfection assays [9]. RFP-RET is constitutively activated without GDNF and GFRa1 [9]. Previously, we developed metallothionein-I (MT)/RFP-RET-transgenic mice of line 304/B6 [10], [11]. Systemic skin melanosis, skin benign melanocytic tumor(s) and skin malignant melanoma(s) developed stepwise in the RET-mice accompanying metastasis to lymph nodes and lungs [10], [11]. These observations suggest that activated RET signaling is correlated with the development of malignant melanoma in mice. However, it remains unknown whether intrinsic c-RET/GDNF signaling is associated with melanomagenesis in RET-mice.

The correlation between c-RET and human malignant melanoma has been denied by a report of no c-RET transcript expression in human cultured-normal melanocytes and malignant melanoma cell lines in Northern blotting analysis [12]. However, a recent study showed that c-RET protein was expressed in human melanomas [13]. These contradictory results indicate that the biological significance of c-RET in malignant melanoma remains unclear. In fact, not only GDNF-mediated c-RET kinase activation but also GFRa1 expression in human malignant melanoma cells has still not been elucidated. In this study, we analyzed c-RET/GDNF signaling in malignant melanoma cells from RET-mice and human cell lines to address the above questions.

## Results

### Tumor stage-dependent RFP-RET transcript expression levels in tumors from RET-mice

**Figure 2:**
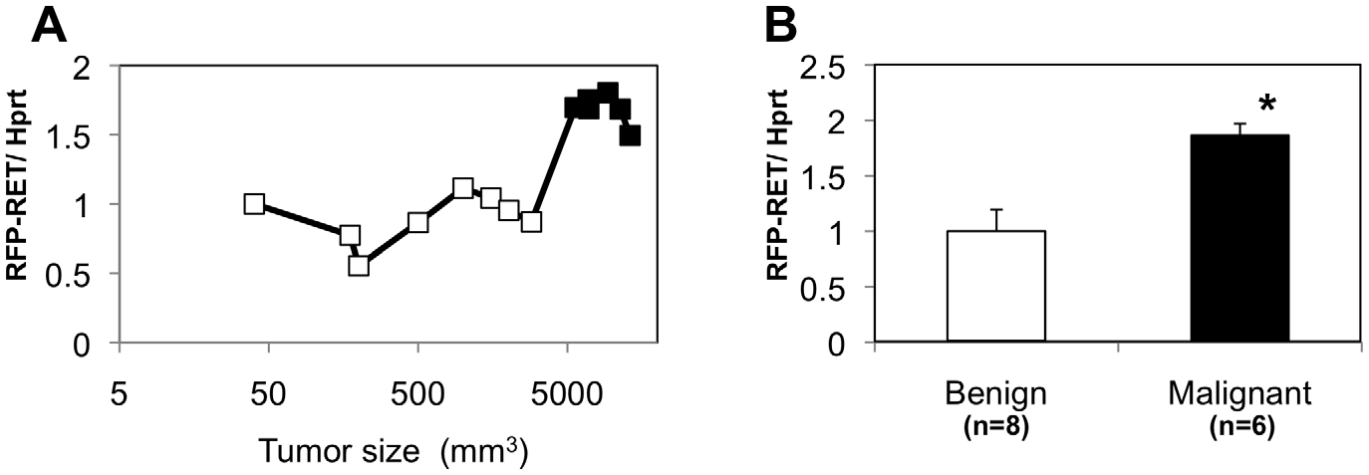
Stage-dependent RFP-RET transcript expression levels in tumors from RET-mice. (A) Levels of RFP-RET transcript expression in tumors of various sizes from RET-mice. Histopathologically benign and malignant tumors are shown by open and closed squares, respectively. (B) Levels of RFP-RET transcript expression (mean ± SD) in benign melanocytic tumors (open bar) and malignant melanoma (closed bar) from RET-mice. RFP-RET transcript levels measured by real-time PCR were adjusted by hypoxanthine guanine phosphoribosyl transferase (Hprt) transcript levels. Difference between expression levels of RFP-RET in benign melanocytic tumors and malignant melanoma from RET-mice was statistically analyzed by the Mann-Whitney *U* test. *, Significantly different (p<0.05) from the control.

We first examined dynamics of the introduced oncogenic RET (RFP-RET: Figure 1B) transcript expression levels in benign and malignant tumors from RET-mice. RFP-RET transcript expression levels were significantly upregulated with increase in tumor size (Figure 2A). In fact, expression levels of RFP-RET transcript in malignant melanoma were about 2-fold higher than those in benign melanocytic tumors (Figure 2B). Results of our previous study using RET-mice also showed that levels of protein expression and activity of RFP-RET in malignant melanoma were increased compared with those in benign melanocytic tumors [10]. These results suggest that constitutively activated RET kinase plays a role in the development of malignant melanoma in mice.

### Tumor stage-dependent expression levels of c-Ret, Gfra1, Gdnf transcripts in tumors from RET-mice

**Figure 3:**
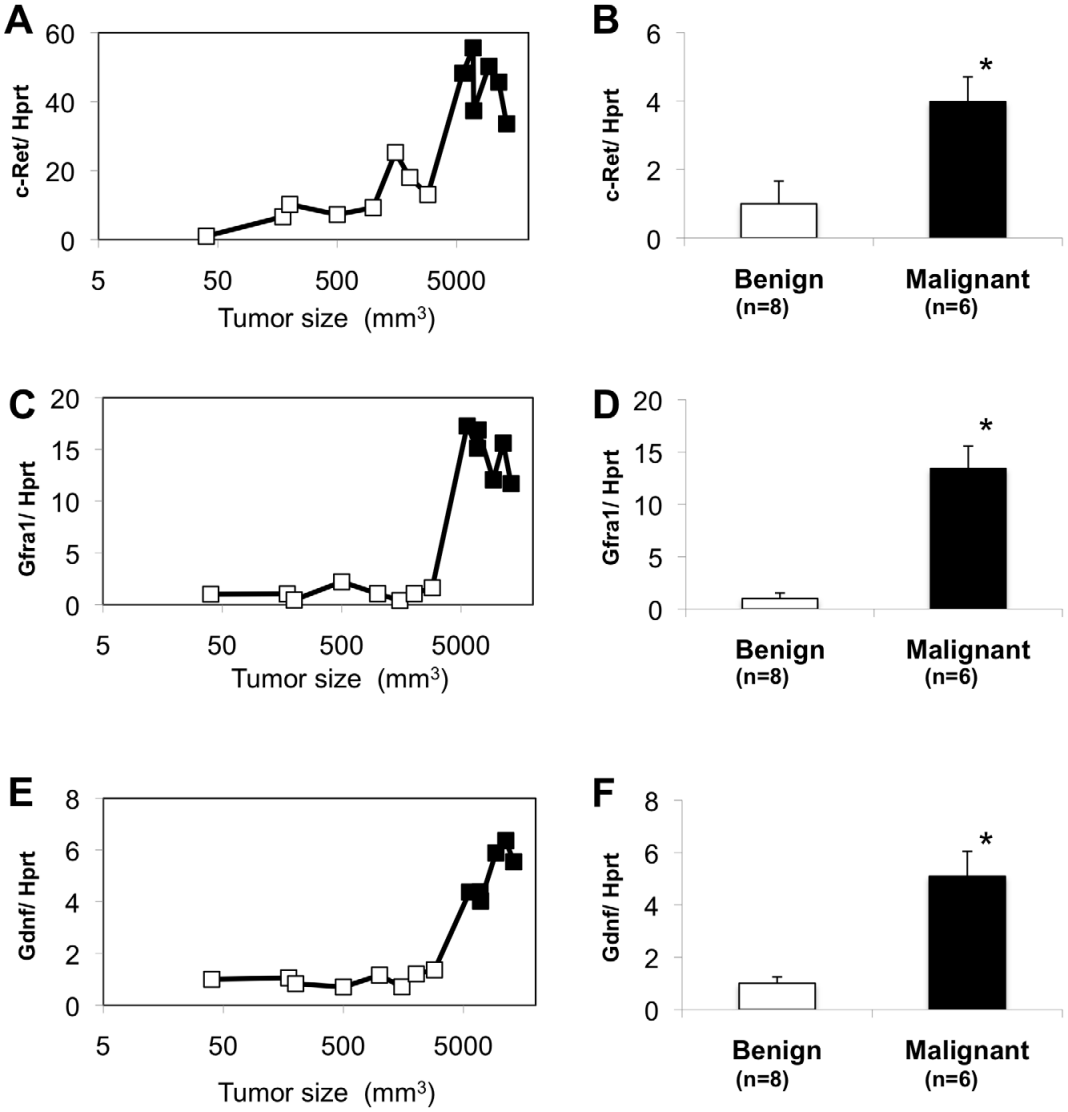
Stage-dependent c-Ret, Gfra1 and Gdnf transcripts expression levels in tumors from RET-mice. (A, C, E) Levels of c-Ret (A), Gfra1 (C) and Gdnf (E) transcripts expression in tumors of various sizes from RET-mice. Histopathologically benign and malignant tumors are shown by open and closed squares, respectively. (B, D, F) Levels of c-Ret (B), Gfra1 (D) and Gdnf (F) transcripts expression (mean ± SD) in benign melanocytic tumors (open bar) and malignant melanoma (closed bar) from RET-mice. c-Ret (A, B), Gfra1 (C, D) and Gdnf (E, F) transcript levels measured by real-time PCR were adjusted by hypoxanthine guanine phosphoribosyl transferase (Hprt) transcript levels. Differences in expression levels of c-Ret (B), Gfra1 (D) and Gdnf (F) between benign melanocytic tumors and malignant melanoma from RET-mice were statistically analyzed by the Mann-Whitney *U* test. *, Significantly different (P<0.05) from the control.

We next examined expression levels of intrinsic c-Ret, Gfra1 and Gdnf transcripts in benign and malignant tumors from RET-mice (Figure 3). Levels of c-Ret transcript expression in malignant melanomas were 4-fold upregulated compared with those in benign melanocytic tumors (Figure 3A, B). The difference between c-Ret transcript expression levels in benign tumors and malignant melanomas from RET-mice was statistically significant (p<0.05; Figure 3B). Gfra1 and Gdnf transcript expression levels in malignant melanomas were also 13-fold and 5-fold upregulated, respectively, compared with those in benign melanocytic tumors (Figure 3C–F). The difference in Gfra1 and Gdnf transcript expression levels between benign tumors and malignant melanomas from RET-mice was statistically significant (p<0.05; Figure 3D, F).

### Expression levels of c-RET, GFRa1, GDNF transcripts in human malignant melanoma cell lines

**Figure 4:**
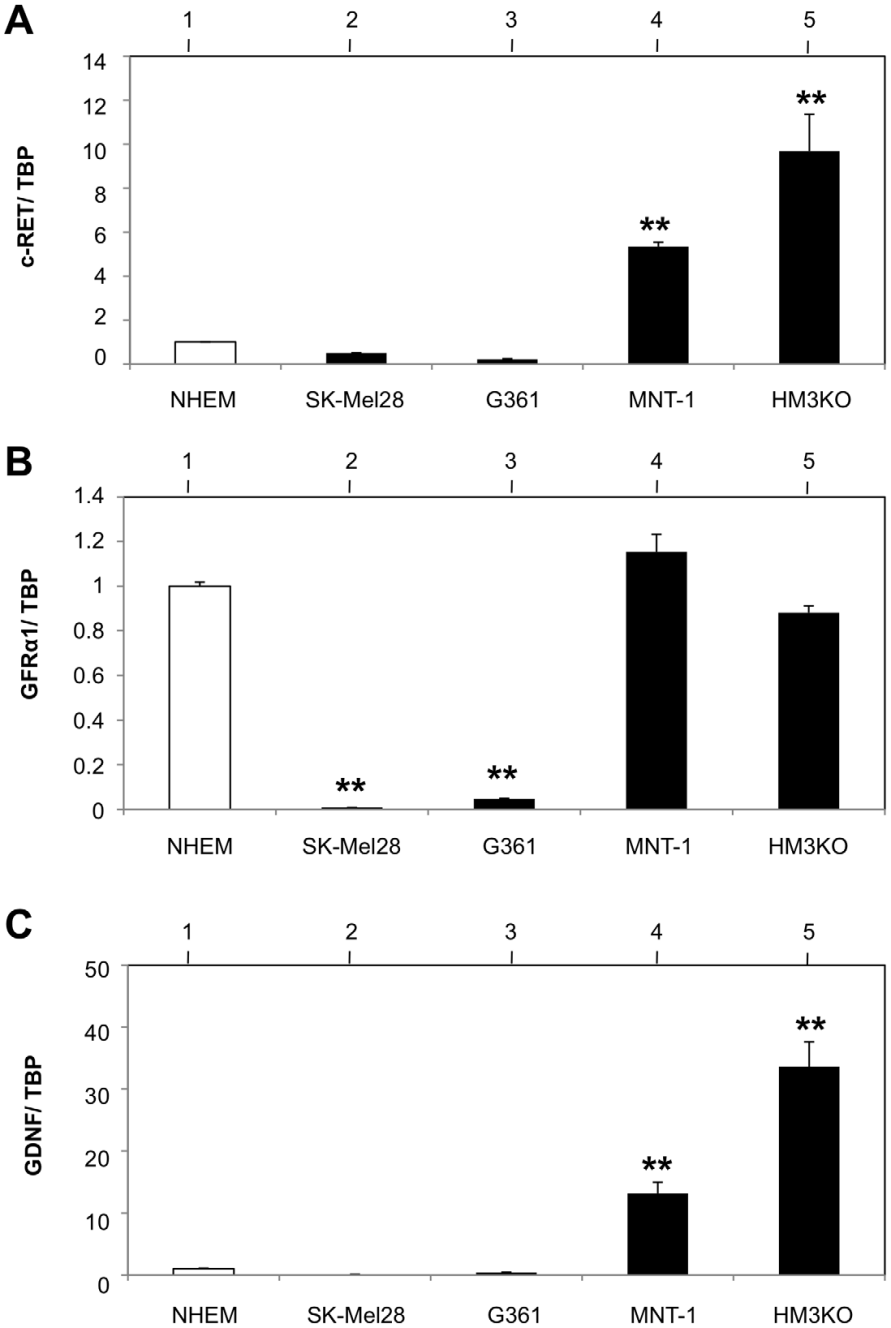
Levels of c-RET, GFRa1 and GDNF transcripts expression in primary-cultured normal human epithelial melanocytes (NHEM) and human malignant melanoma cell lines. (A, B, C) Levels of c-RET (A), GFRa1 (B) and GDNF (C) transcripts expression in NHEM (lane 1) and 4 kinds of malignant melanoma cell lines (lanes 2–5; SK-Mel28, G361, MNT-1 and HM3KO). The transcript levels measured by real-time PCR were adjusted by TATA-box-binding protein (TBP) transcript levels. Differences in expression levels of c-Ret (A), Gfra1 (B) and Gdnf (C) between NHEM (lane 1; open bar) and malignant melanoma cell lines (lanes 2–5; closed bars) were statistically analyzed by the Kruskal-Wallis test. **, Significantly different (P<0.01) from the control.

We next examined the expression levels of c-RET, GFRa1 and GDNF in primary-cultured normal human epithelial melanocytes and human malignant melanoma cell lines (G361, SK-Mel28, MNT-1 and HM3KO) [14]–[16]. Transcript expression levels of c-RET and GDNF in MNT-1 cells were around 5-fold and 12-fold upregulated, respectively, compared with those in NHEM cells (Figure 4A, C). Transcript expression levels of c-RET and GDNF in HM3KO cells were around 10-fold and 35-fold increased, respectively, compared with those in NHEM cells (Figure 4A, C). There were no differences in GFRa1 transcript expression levels among NHEM, MNT-1 and HM3KO cells (Figure 4B). The expression levels of c-RET, GFRa1 and GDNF in G361 and SK-Mel28 human malignant melanoma cells were definitely lower or undetectably low compared with those in NHEM cells (Figure 4A–C).

### Levels of c-RET and GFRa1 protein expression in human melanocytic cells

**Figure 5 pone-0010279-g005:**
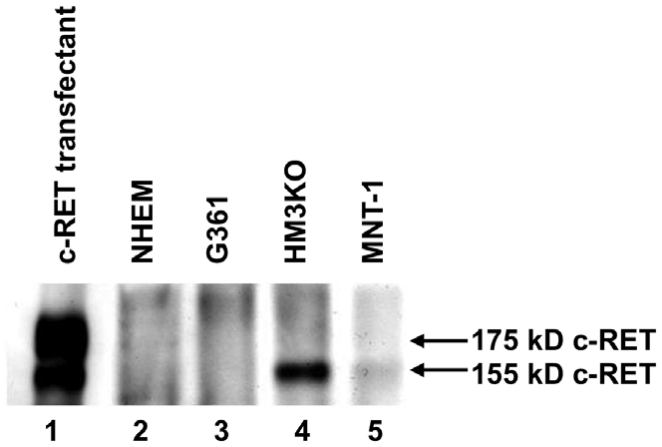
Levels of c-RET protein expression in human malignant melanoma cell lines. The levels of c-RET protein expression were examined in c-RET-transfected NIH3T3 cells as a positive control (c-RET transfectant; lane 1), primary-cultured normal human epithelial melanocytes (NHEM; lane 2), G361 (lane 3), HM3KO (lane 4) and MNT-1 (lane 5) by immunoblotting analysis with anti-RET antibody after immunoprecipitation with anti-RET antibody.

**Figure 6 pone-0010279-g006:**
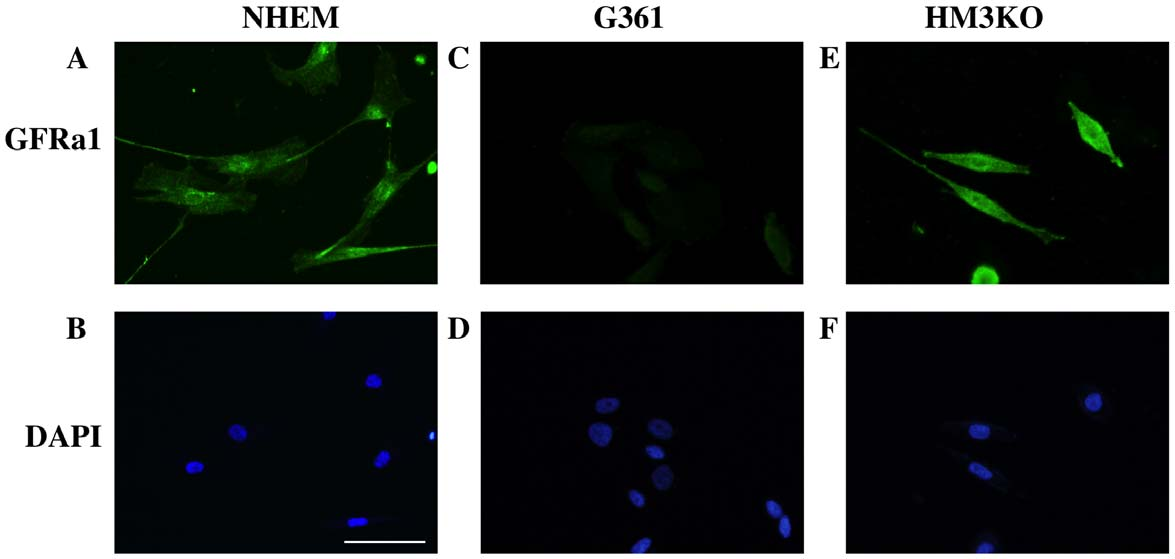
GFRa1 protein expression in human melanocytic cells. GFRa1 protein expression was examined in primary-cultured normal human epithelial melanocytes (NHEM; A, B), G361 cells (C, D) and HM3KO cells (E, F) by immunocytochemistry with anti-GFRa1 antibody (A, C, E) using DAPI counterstaining (B, D, F).

We next examined levels of c-RET protein expression in NHEM and human malignant melanoma cells (G361, HM3KO and MNT-1) (lanes 2–5 in Figure 5). Expression of 155-kD c-RET protein was detected in HM3KO and MNT-1 cells (lanes 4 and 5 in Figure 5) but not in NHEM and G361 cells (lanes 2 and 3 in Figure 5), though the level of c-RET protein expression in MNT-1 cells was weak. After selecting HM3KO as a highly expressing c-RET cell line and G361 as a barely expressing c-RET cell line, we confirmed GFRa1 protein expression in the cells. NHEM (Figure 6A, B) and HM3KO cells (Figure 6E, F), but not G361 cells (Figure 6C, D), expressed GFRa1 protein. These results for c-RET (Figure 5) and GFRa1 (Figure 6) protein expression in NHEM, G361 and HM3KO cells correspond to c-RET and GFRa1 transcript expression (Figure 4A, B).

### c-RET/GDNF signaling in human malignant melanoma cells

**Figure 7 pone-0010279-g007:**
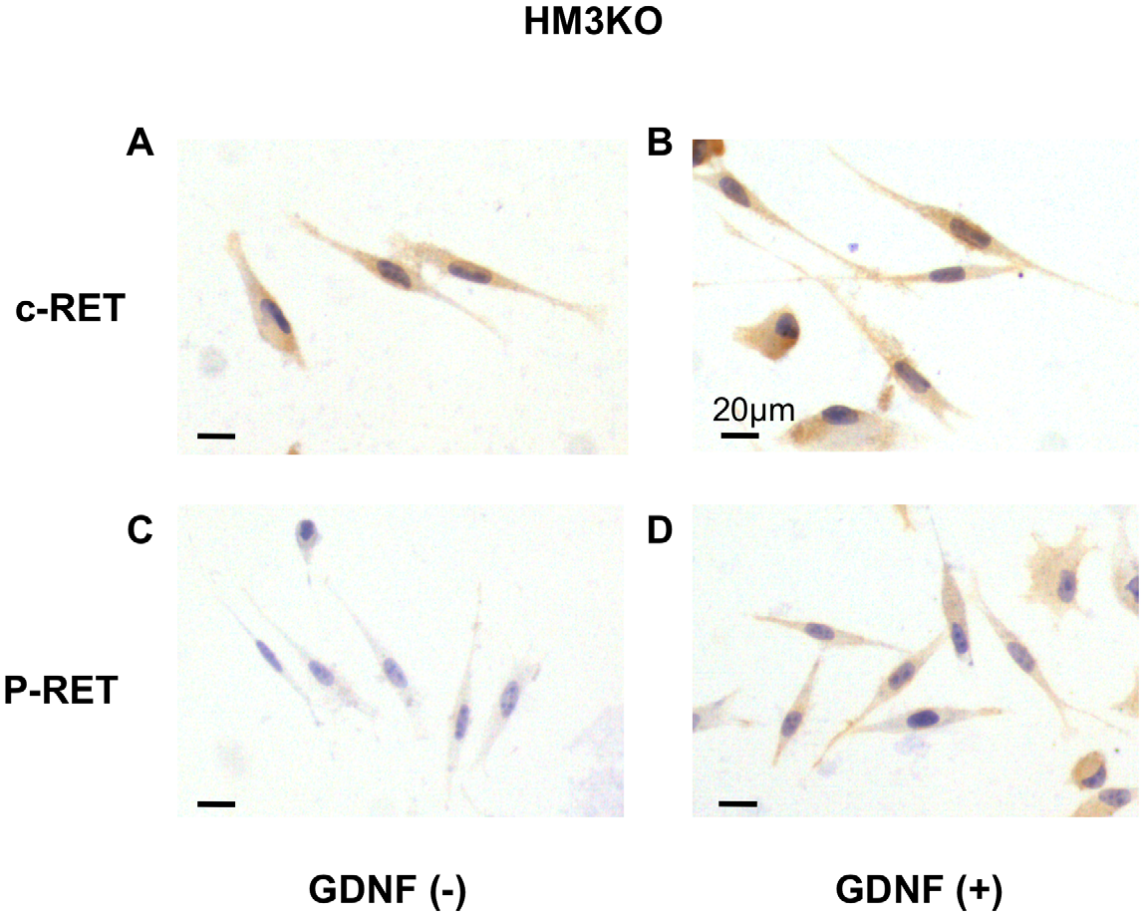
Augmentation of c-RET tyrosine kinase activity in HM3KO cells by c-RET ligand (GDNF). Expression of c-RET protein (A, B) and phosphorylated tyrosine 905 in c-RET (C, D) in HM3KO cells in the absence (A, C) or presence (B, D) of GDNF were examined by immunocytochemistry with anti-c-RET and anti-phosphorylated tyrosine 905 in c-RET antibodies using hematoxylin counterstaining (A–D).

**Figure 8 pone-0010279-g008:**
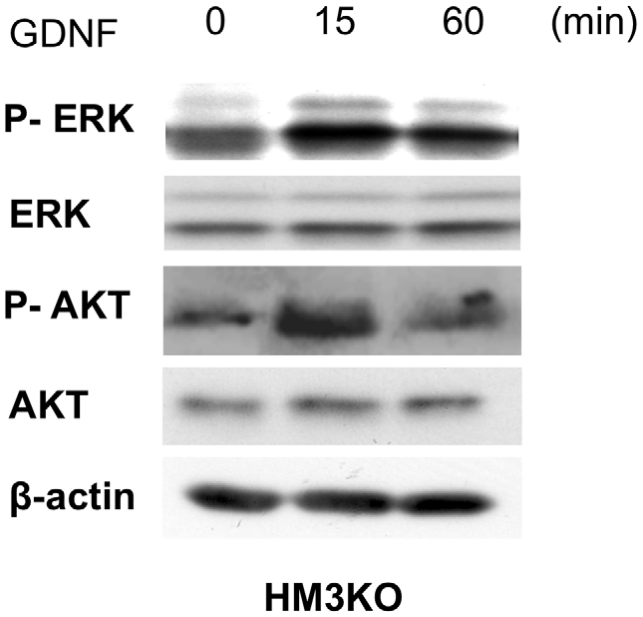
Signal transduction molecules potentially sited downstream of c-RET in HM3KO cells. Expression and phosphorylation levels of ERK and AKT in HM3KO cells before (0 min) and at 15 and 60 min after stimulation with GDNF (100 ng/ml) were examined by immunoblotting. Equality of protein amounts in each lane was confirmed by immunoblotting with anti-β-actin antibody.

Since both c-RET and GFRa1 were found to be expressed in HM3KO cells (Figures 4–6), we next examined whether c-RET tyrosine kinase in HM3KO cells is activated by its ligand (GDNF). Not only biochemical analysis (lane 4 in Figure 5) but also immunocytochemical analysis (Figure 7A, B) revealed that c-RET protein was expressed in HM3KO cells. Phosphorylation of tyrosine 905 in HM3KO cells was increased by GDNF (Figure 7C, D). On the other hand, immunocytochemical analysis also revealed that there was no c-RET protein expression and no augmentation of c-RET kinase activity by GDNF in G361 cells without c-RET protein expression (data not shown), partially in accordance with the previous results (lane 3 in Figure 5). These results suggest that c-RET/GDNF signaling worked in HM3KO cells but not in G361 cells. We next examined whether signal transduction molecules potentially sited downstream of c-RET [2], [3], [6] in HM3KO cells are activated by GDNF. Phosphorylation levels of ERK and AKT in HM3KO cells (Figure 8), but not in G361 cells (data not shown), were increased 15 min after stimulation with GDNF, while expression levels of ERK, AKT and ß-actin proteins were comparable.

### Augmented proliferation of human malignant melanoma cells by GDNF stimulation

**Figure 9:**
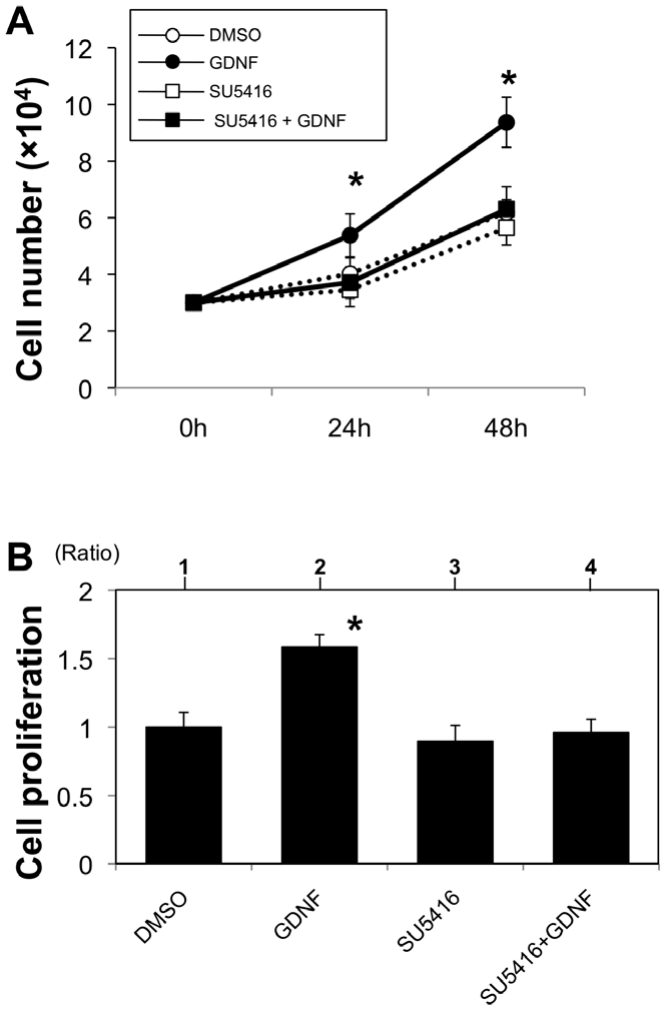
Proliferation of HM3KO cells. Proliferation of HM3KO cells treated with a solvent (0.1% of DMSO) (open circle in A, lane 1 in B), GDNF (100 ng/ml) (closed circle in A, lane 2 in B), SU5416 (5µM) (open square in A, lane 3 in B) and GDNF plus SU5416 (closed square in A, lane 4 in B) for 24 (A) and 48 hours (A, B) was examined by cell counting with trypan blue staining (A) and MTT assay (B). Difference between proliferation levels of DMSO-treated control cells and other cells was statistically analyzed by the Kruskal-Wallis test. *, Significantly different (P<0.05) from the control.

We finally examined the physiological effect of GDNF stimulation on proliferation of HM3KO and G361 human malignant melanoma cells, which have no polymorphism at the G691S juxtamembrane region in c-RET as shown in a previous study [13]. GDNF increased the number of HM3KO cells (Figure 9A) but not the number of G361 cells (data not shown). MTT assay also showed significant proliferation of GDNF-stimulated HM3KO cells (Figure 9B) but not G361 cells (data not shown). GDNF-stimulated proliferation of HM3KO cells, but not that of G361 cells (data not shown), was suppressed by the RET kinase inhibitor SU5416 [17] (Figure 9A and B).

## Discussion

We previously demonstrated that cutaneous malignant melanomas develop in oncogenic RET (RFP-RET)-carrying transgenic mice [10], [11]. Our previous and present results showed that constitutively activated RFP-RET increased RFP-RET protein expression [10], [18] and transcript (Figure 2) expression levels in the process of melanomagensis in the RET-mice. On the other hand, the present results showed that not only RFP-RET but also c-Ret, Gfra1 and Gdnf expression levels in malignant melanomas were definitely increased compared with those in benign melanocytic tumors (Figures 2, 3) in RET-mice. Since continuously activated RET kinase increased the levels of RET transcript and protein expression [19], these results suggest that constitutively activated RFP-RET enhances intrinsic c-RET protein expression in the process of melanomagensis in the RET-mice. These dynamics of intrinsic c-Ret/Gfra1/Gdnf and introduced RFP-RET signaling in melanocytic tumors from RET-mice encourage us to examine the correlation between RET and human malignant melanoma.

Previous studies showed that overexpression of c-RET, GFRa1 and/or GDNF indicated poor prognosis in human pancreatic [20] and bile duct [21] carcinomas. Overexpression of GDNF was also reported to be involved in tumorigenesis of human lung cancer [22]. A recent study has biochemically provided evidence that c-RET/GDNF signaling promotes proliferation of human breast carcinoma cells [23]. These results suggest that c-RET/GDNF signaling is correlated with the pathogenesis of human cancers. In this study, we examined whether c-RET/GNDF signaling worked in human malignant melanoma cells. Our results showed that transcript expression levels of c-RET and GDNF in MNT-1 and HM3KO human malignant melanoma were definitely higher than those in NHEM cells, while GFRa1 expression levels were comparable in these cells (Figure 4). Further analysis biochemically (Figure 5) and immunohistochemically (Figure 7) revealed that c-RET protein was expressed in HM3KO cells but not in NHEM and G361 cells. As shown in a previous study [13], a 155-kDa c-RET protein, but not a 175-kDa c-RET protein, was detected in our study (Figure 5). Then we showed that transcript and protein of GFRa1, which is essential for c-RET/GDNF signaling, was expressed in malignant melanoma cells by real-time PCR (Figure 4) and immunohistochemistry (Figure 6). We also detected for the first time increased phosphorylated levels of tyrosine 905 in c-RET in HM3KO cells by immunohistochemical analysis (Figure 7), although we failed to detect increased phosphorylated levels by immunoblot analysis. Thus, we further showed GFRa1 expression and GDNF-mediated phosphorylation of c-RET kinase in human melanoma cells (Figures 4–7) in addition to the recent report showing a correlation between RET and human melanoma [13]. We next showed that signal transduction molecules (ERK and AKT) potentially sited downstream of c-RET were activated by GDNF in HM3KO cells (Figures 8). These results suggest that GDNF stimulates both RET-RAS-RAF-ERK and RET-phosphatidylinositol 3-kinase (PI3K)-Akt pathways in HM3KO cells. We finally showed that a c-RET agonist (GDNF) promoted cell proliferation and that a c-RET antagonist (SU5416) [17] inhibited proliferation of HM3KO cells (Figure 9). These results suggest that c-RET/GFRa1/GDNF signaling plays a role in proliferation of HM3KO human malignant melanoma cells. Furthermore, a previous report revealed that GDNF stimulation significantly amplified proliferation of human melanoma cells with polymorphism at G691S in c-RET [13]. Our results presented in Figure 9 newly showed that GDNF stimulation also amplifies the proliferation of HM3KO human malignant melanoma cells without the polymorphism.

In summary, we newly showed c-RET protein expression in HM3KO and MNT-1 melanoma cells in addition to its expression in the previously reported five human melanoma cell lines [13]. Moreover, we for the first time demonstrated not only GFRa1 protein expression but also GDNF-mediated c-RET kinase activation via phosphorylated tyrosine 905 in human malignant melanoma cells. Our results for activated RET and signal transduction molecules in human melanoma cells (Figures 4–9) partially correspond to our previous reports of increased activation and protein expression levels of RET and signal transduction molecules sited downstream in the process of melanomagenesis in RET-mice [10], [18]. Thus, we might have partially addressed the correlation between RET and malignant melanoma by integrating the previous and present results for mice and humans.

## Materials and Methods

### Mice

We previously established RET-mice (line 304/B6) with C57BL/6 background by introducing the *RET* oncogene (RFP-RET) fused to the mouse MT promoter-enhancer [10]. All mice were kept on a 12-h light-dark cycle in a temperature- and humidity-controlled environment in the Animal Research Center of Chubu University. This study was formally approved by Chubu University (approval no.: 2010001)

### Real-time PCR

Total RNA was prepared from frozen tumor and human cell line samples using a High Pure RNA Kit (Roche Diagnostics) according to the method previously described [24]. cDNA was then synthesized by reverse transcription of total RNA using Supercript™III reverse transcriptase included in the RT enzyme mix and RT reaction mix according to the protocol of the manufacturer (Invitrogen). Real-time quantitative RT-PCR with SYBR green was performed using power SYBR® Green PCR master mix (Applied Biosystems) in an ABI Prism7500 sequence detection system (Applied Biosystems). The expression levels of c-RET, GFRa1 and GDNF transcripts measured by quantitative RT-PCR (real-time PCR) were adjusted by the transcript expression level of TATA-box-binding protein (TBP) for human samples or hypoxanthine guanine phosphoribosyl transferase (Hprt) for mice samples. The following pairs of forward and reverse primers were prepared for amplification: mice c-Ret, 5′-GCTGCATGAGAATGACTGGA-3′ and 5′-TGGCATfTCTCCCTCTCTCTG-3′ (PCR product size, 177 bp); mice Gfra1, 5′-GACCTGGAAGATTGCCTGAA-3′ and 5′-CAGTGGTAGTCGTGGCAGTG-3′ (PCR product size, 148 bp); mice Gdnf, 5′-GTCCAACTGGGGGTCTACG-3′ and 5′-AGCAACACCAGGCAGACAG-3′ (PCR product size,101 bp); mice Hprt, 5′-CTTTGCTGACCTGCTGGATT-3′ and 5′-TATGTCCCCCGTTGACTGAT-3′ (PCR product size, 121 bp); human RFP-RET, 5′-TGACGGAGAGTCTAAAGCAG-3′ and 5′-GCTTTAATCCCCCGGGGC-3′ (PCR product size, 139 bp); human c-RET, 5′-GCTCCACTTCAACGTGTC -3′ and 5′-GCAGCTTGTACTGGACGTT-3′ (PCR product size, 158 bp); human GFRa1, 5′-CACTGCCACTACCACCACTG-3′ and 5′-GTGTATTGCCCGACACATTG-3′ (PCR product size, 146 bp); human GDNF, 5′-CTGGGCTATGAAACCAAGGA-3′ and 5′-CAACATGCCTGCCCTACTTT-3′ (PCR product size, 143 bp); human TBP, 5′-CACGAACCACGGCACTGATT-3′ and 5′-TTTTCTTGCTGCCAGTCTGGAC-3′ (PCR product size, 89 bp). PCR was carried out using 10 µl of power SYBR® Green PCR master mix (Applied Biosystems) containing 5 µM forward primer and 5 µM reverse primer in a final volume of 20 µl. The PCR conditions were as follows: 50°C for 2 min, 95°C for 10 min and 40 cycles of 95°C for 15 s and 60°C for 1 min.

### Analysis for polymorphism at the G691S juxtamembrane region in c-RET

Polymorphism at the G691S juxtamembrane region in c-RET in melanoma cells was examined according to the method previously reported [13].

### Immunoprecipitation and Immunoblotting

Immunoprecipitation with anti-c-RET rabbit polyclonal antibody was performed by the method previously described [25]. Immunoblotting with anti-c-RET (#18128, IBL, Fujioka, Japan), anti-ERK (#9102 Cell Signaling, MA), anti-phosphorylated ERK (#E7028 SIGMA), anti-AKT (#9272 Cell Signaling, MA) and anti-phosphorylated AKT (#9271 Cell Signaling, MA) antibodies was performed according to the method described previously [25].

### Immunocytochemistry

G361 and HM3KO cells were treated with 100 ng/ml GDNF for 15 minutes at 37°C, and then the cells were fixed in freshly prepared phosphate-buffered 2% paraformaldehyde for 15 min at 4°C. For DAB staining, the fixed cells were incubated in 3% hydrogen peroxide for 10 min at RT. All specimens were treated with a blocking reagent (phosphate-buffered 1% BSA (fraction V) solution containing 0.2% gelatin and 0.05% tween20) and then incubated with the following primary antibodies for 60 min at room temperature: anti-GFRa1 (H-70, Santa Cruz Biotechnology, CA), anti-c-RET (#18128, IBL, Fujioka, Japan) and anti-phosphorylated tyrosine 905 in c-RET (#3221, Cell Signaling, MA) antibodies. Then the cells were stained using a DAKO Envision-HRP/DAB system (K1390) (DAKO, CA) or labeled with Alexa fluore 488-conjugated donkey anti-rabbit IgG (A21206) (Invitrogen, OR). Counterstaining was performed using hematoxylin or DAPI.

### Cell proliferation

Cell proliferation in the presence or absence of GDNF was evaluated by counting cells with trypan blue staining [26] and by 3-(4,5-dimethylthiazol-2-yl)-2,5-diphenyltetrazolium bromide (MTT) assay following previous methods [27].

### Cell lines

Normal human normal melanocytes (NHEM) were purchased from Cell Applications Inc, and were maintained with melanocyte growth medium containing hydrocortisone and growth supplements (Cell Applications Inc.). MNT-1 and HM3KO human malignant melanoma cell lines were kindly provided by Dr. Tamio Suzuki (Department of Dermatology, Yamagata University School of Medicine, Yamagata, Japan). G361 cells were kindly provided by Cell Resource Center for Biomedical Research, Tohoku University. SK-Mel28 cells were purchased from Riken Bioresource Center Cell Bank. SK-Mel28, MNT-1 and HM3KO cells were maintained in Dulbecco's Modified Eagle's Medium (DMEM) supplemented with 10% fetal bovine serum (FBS). Other cell lines were maintained in RPMI 1640 supplemented with 10% FBS.
